# Swimming Training Prevents Stress‐Induced Spatial Memory Impairment by Reducing Neurodegenerative Changes in Hippocampus

**DOI:** 10.1155/bri/9980466

**Published:** 2025-12-30

**Authors:** Mohammad Amin Safari, Maryam Koushkie Jahromi, Hadi Aligholi, Rasoul Rezaei, Zahra Zeraatpisheh, Parisa Foroozan, Atiyeh Separdanasab

**Affiliations:** ^1^ Department of Sport Sciences, School of Education and Psychology, Shiraz University, Shiraz, Iran, shirazu.ac.ir; ^2^ Department of Neuroscience, School of Advanced Medical Sciences and Technologies, Shiraz University of Medical Sciences, Shiraz, Iran, sums.ac.ir; ^3^ Biotechnology Research Center, Shiraz University of Medical Sciences, Shiraz, Iran, sums.ac.ir; ^4^ Research Center for Psychiatry and Behavioral Sciences, Shiraz University of Medical Sciences, Shiraz, Iran, sums.ac.ir

**Keywords:** astrogliosis, dark neurons, recovery, spatial memory, stress, swimming

## Abstract

This study investigated the effects of swimming training (ST) on hippocampal structure, specifically glial fibrillary acidic protein (GFAP), dark neurons, and the thickness of the CA1 region and dentate gyrus (DG), as well as spatial memory performance in young male rats subjected to chronic stress (CS). Adult male Wistar rats were randomly assigned to five groups: swimming training (ST), chronic mild stress (CMS), chronic mild stress followed by swimming training (CS + ST), chronic mild stress followed by a recovery period (CS + recovery), and a control group with no exercise or stress intervention. Spatial memory was assessed using the Morris water maze (MWM). The findings revealed that the ST group exhibited the lowest levels of GFAP and dark neurons, as well as the highest thickness of the CA1 and DG regions and the best spatial memory performance. In the CS + ST group, ST reduced GFAP and dark neurons, increased the thickness of the CA1 and DG, and improved memory performance compared to the control and CS + recovery groups. The CS group had the highest levels of GFAP and dark neurons, alongside the thinnest CA1 and DG regions. Meanwhile, in the CS + recovery group, GFAP and the number of dark neurons were lower than in the CS group. In summary, ST reduced GFAP and the number of dark neurons in both stressed and nonstressed rats, with a more pronounced effect observed in nonstressed rats. Overall, ST improved stress‐induced spatial memory performance and hippocampal morphology in young male rats.

## 1. Introduction

Repeated and unpredictable stress are associated with unfavorable psychological and physical health issues [1]. When environmental, physical, and psychological stressors exceed, individual coping strategies in the middle and long‐term physiological homeostasis are impaired. This can result in increased production or inadequate removal of free radicals, leading to irreversible cellular degradation [2], which can then impair neural morphology and function.

In the case of chronic psychological stress, higher cortisol and inflammatory concentrations appear to be associated with a smaller hippocampal volume [3]. Stress may affect the hippocampus through the hyperactivity of the hypothalamic–pituitary–adrenocortical (HPA) axis. The key product of the HPA axis is cortisol [4, 5]. Thus, altering the HPA axis activity appears to be causally associated with atrophic changes in the hippocampus region [6, 7].

The hippocampus is the main neuronal structure for spatial memory [8, 9]. Long‐term spatial memory is defined as stable changes in spatial information processing over time. This information processing consists of encoding, consolidation, storage, and retrieval of specific spatial information [10]. Stress can impact memory affecting the endocrine system [11] and various regions of the hippocampus [12]. Chronic stress (CS) can increase glial fibrillary acidic protein (GFAP) as an intermediate filament‐III protein uniquely found in astrocytes in the CNS, nonmyelinating Schwann cells in the peripheral nervous system (PNS), and enteric glial cells [13] and increase dark neurons as contracted, intensely stained neurons in the hippocampus. Dark neurons are a histological hallmark of neuronal injury, characterized by shrunken, hyperbasophilic somata and corkscrew dendrites. Their formation is often associated with excitotoxicity, metabolic stress, and cytoskeletal collapse, making them a sensitive indicator of early neurodegenerative changes [14]. Stress can be effective on the formation of dark neurons [15]. Therefore, quantifying dark neurons provides a crucial morphological correlate to functional memory deficits specially in physical and mental stress conditions.

While physical activity appears to favorably impact glucocorticoid secretion and the sensitivity of their receptors [16], long‐term exercise has the potential to favorably impact the stress response in humans [17]. Physical activity can induce structural and functional plasticity in the hippocampus [18], instigate neurogenesis [19], increase the volume of the hippocampus [20, 21], and favor angiogenesis and neuronal plasticity [18]. Furthermore, physical exercise appears to favorably impact adult hippocampal neurogenesis. In rats, exercise enhances long‐term potentiation (LTP) in the dentate gyrus (DG) and robustly improves spatial learning and memory performance [22–25] as well as ameliorating memory deficits [26]. Exercise may increase the expression of GFAP in astrocytes [27] and alter the morphology of astrocytes via the reduction of the ramification and length of astrocytes [28, 29]. Thus, as possible mechanisms related to the effect of exercise on memory, we investigated them in the present study. Furthermore, various types of exercises may induce different effects on neural and memory adaptations [30].

Swimming is one of the exercises associated with (1) increased secretion of endorphins from the brain, (2) an increase in the feeling of pleasure [31]; (3) secretion of antioxidant enzymes [31], (4) neurogenesis in the hippocampus [32], (5) decreased corticosterone levels, and (6) improved cognitive behaviors in stress‐induced rats [33]. However, the effect of swimming as a rehabilitative intervention and on the morphology and function of the hippocampus in previously induced stress conditions in humans or animals is not clear yet. Given this background, the question arises; does swimming training at a constant intensity affect both the morphology of the hippocampus and long‐term memory performance in young male rats exposed to CS?

## 2. Methods

### 2.1. Animals and Procedures

In this experimental study, a total of 40 three‐month‐old male Wistar rats were used. The rats were kept under normal light conditions (12 h light/dark cycle) in laboratory cages (3 in each cage) at a temperature of 25 ± 2°C to comply with the test conditions. Water and food (consisting of a total of 16.6 kJ/g: carbohydrate 66.40%, fat 10.60%, and protein 23%) were freely available. Animal inclusion criteria, considered before providing animals, were age, sex, and health of the rats, as confirmed by a veterinarian. Exclusion criteria were animals suffering from disease or not performing exercise during the intervention period. The number of animals in each group was determined according to the statistical test and the number of groups [34], which indicated that a total sample size of approximately 30 animals (*n* = 6 per group) would provide sufficient statistical power for the planned analyses (one‐way analysis of variance (ANOVA)). To account for potential attrition due to the stress protocol or an inability to adapt to the swimming intervention, a larger number of animals were initially recruited. Specifically, eight rats were randomly assigned to groups that included swimming training (to anticipate exclusions due to poor swimming adaptation), and seven rats were assigned to the CS group (to anticipate potential mortality from stress). The remaining groups were assigned six rats each. During the study, two rats from the swimming groups (one from ST and one from CS + ST) were excluded as they failed to successfully adapt to the swimming protocol, defined as an inability to swim for the prescribed duration despite the gradual adaptation period, showing signs of extreme distress or fatigue (e.g., excessive floating, sinking, or frantic struggling). One rat in the CS group died during the stress protocol.

To maintain equal group sizes (*n* = 6) for a balanced statistical analysis, one additional rat from the CS group was randomly excluded from the dataset. Consequently, the final analysis was performed with six rats in each of the five groups (*N* = 30).

After 1 week of maintenance in the laboratory, the rats were divided into the following five study groups: CON: no treatment for 10 weeks; CS + ST: First: 3 weeks of stress; second, followed by 2 weeks of adaptation with water and swimming; third, followed by 4 weeks of swimming; ST: First: 2 weeks of adaptation with water and swimming, second, followed by 4 weeks of swimming; CS: 3 weeks of stress; CS + recovery: First, 3 weeks of stress; second, followed by 6 weeks of no further intervention.

**Figure 1:**
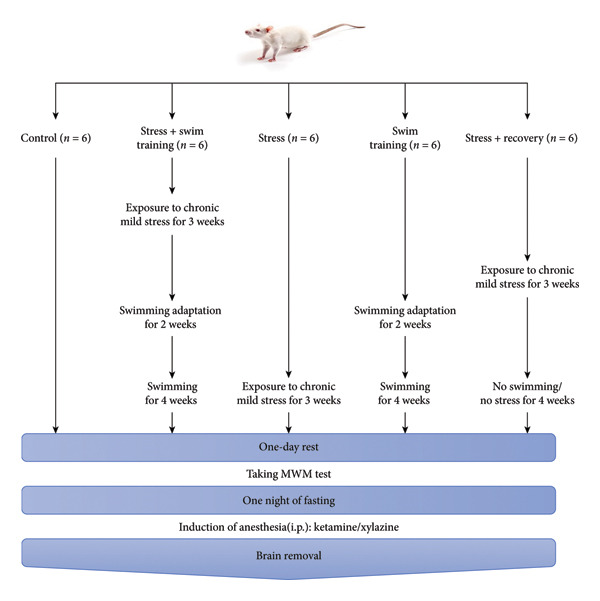
Schematic view of experimental procedures.

To measure memory, 24 h after each intervention, the Morris water maze (MWM) test was performed. Finally, six rats in each group received cardiac perfusion after anesthesia with ketamine xylazine. Then, their brains were removed and fixed in 10% formalin; after 24 h, the tissues were sent to the laboratory for the measurement of astrogliosis, dark neurons, thickness of CA1, and DG region of the hippocampus. To control the possible effect of age on the studied variables, the intervention schedule was adjusted so that all rats were euthanized at the same age and time frame. Measurements were performed by specialists who were blinded about the study outcomes. The summary of the study protocol is presented in Figure 1.

### 2.2. Ethical Approval

The study was conducted in accordance with the Declaration of Helsinki and approved by the ethics committee of the Shiraz University of Medical Sciences (Approval code: IR.SUMS.AEC.1400.034).

### 2.3. Note on Cohort Reuse

The same cohort of animals (*N* = 40, final *N* = 30) was used in a prior publication [35], where the focus was on anxiety‐like behaviors (open field, elevated plus maze), serum corticosterone, and body weight dynamics. In contrast, all histological (GFAP, dark neurons, hippocampal layer thickness) and spatial memory (MWM performance) data reported here are novel and were not analyzed, reported, or published in that earlier work. This reuse aligns with the 3Rs principle (Reduction) and ensures maximal scientific yield from a single ethically approved cohort.

### 2.4. Swimming Training

Animals in the swimming groups performed 60 min of swimming per day, for 5 days a week, and for 4 consecutive weeks [35].

To control the effect of the environment, sedentary (nonexercise) rats were placed in shallow water for 30 min a day at a temperature of 31°C and were under controlled conditions [36]. To reduce stress without promoting physical training adaptations, all rats were adapted to water before initiating the main swimming program. This adaptation consisted of two parts. The first part focused on adaptation to water, which was performed for 4 days. On the first day, the animals were placed for 5 min into the swimming pool with shallow water in which they could stand. On the second day of adaptation, rats were placed for 5 min in head‐high water, in which they could start swimming. On the third day, the water was deep enough, so that they had to swim for 5 min. On the fourth and last day of adaptation, the animals had to swim for 15 min. The water temperature was maintained at 31°C [33]. The second part of the protocol focused on rat adaptation to the swimming exercise, which was performed for 6 days and began with 15 min of swimming. The second, third, fourth, fifth, and sixth days of adaptation consisted of 24, 33, 51, and 60 min of swimming exercise, respectively [35].

### 2.5. Chronic Mild Stress (CMS) Protocol

The CMS protocol was administered for 3 weeks based on established models. This protocol consisted of exposing rats to a variety of unpredictable, mild stressors to prevent habituation. Rats were exposed to CMS for 21 days, including 2 h of paired caging, 18 h of free access to food followed by 1.5 h of restricted access to food (0.2 g pellet), 18 h of water deprivation followed by 1.5 h of empty bottle exposure, 21 h of wet cage (300 mL of water added per 100 g of bedding), 36 h of continuous exposure to light, and 3 h of 45° cage tilting. These stressors were applied in a random order each day over a 7 day cycle, which was then repeated for the 3 week duration [6]. The detailed weekly schedule is presented in Table 1.

**Table 1:** Weekly schedule of the chronic mild stress (CMS) protocol.

Day	Time	Stressor and duration
Sunday	16:00 ⟶ Monday 10:00	Food deprivation (18 h)
Monday	07:00 ⟶ Tuesday 19:00	Overnight illumination (36 h)
	10:00–11:30	Food restriction (1.5 h)
Tuesday	17:00 ⟶ Wednesday 14:00	Wet cage (21 h)
Wednesday	11:00–14:00	Tilted cage (3 h)
	18:00 ⟶ Thursday 12:00	Water deprivation (18 h)
Thursday	08:00–09:30	Empty bottle exposure (1.5 h)
	12:00–14:00	Paired housing (2 h)
Friday	07:00 ⟶ Saturday 04:00	Water deprivation (21 h)
Saturday	07:00–08:30	Empty bottle exposure (1.5 h)

### 2.6. MWM Test

**Figure 2:**
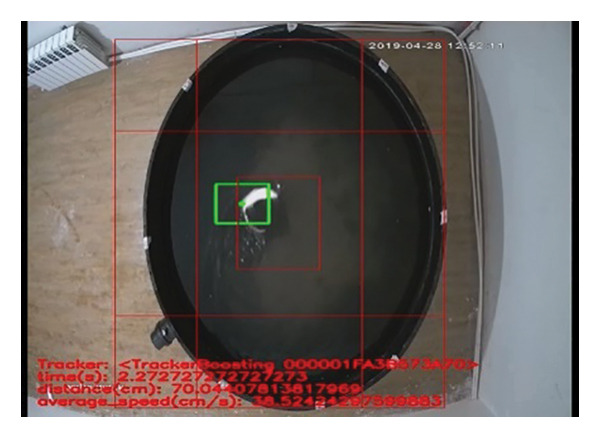
Morris water maze test performance tool.

The MWM test, consisting of 3–6 consecutive days of oriented navigation trials and a 60 s of probe trial, was performed to evaluate spatial memory as described previously​ [37] with some modifications. The water maze was a black circular pool (150 cm in diameter, 50 cm in height) filled with opaque water at 23 ± 1°C and surrounded by various visual cues. Moreover, the tank was divided into 4 equal quadrants (marked, respectively, as E, S, W, and N) with two imaginary perpendicular lines. During the oriented navigation trial, each rat participated in four trials per day to learn locating the platform (9 cm in diameter) that was fixed in the quadrant E and submerged 1.5 cm underneath the surface of water. The animal was gently placed into the pool at the midpoint of the edge of the quadrant, changed for each trial, and was allowed a maximum of 120 s to locate the platform. Rats failing to find the platform within 120 s were guided toward it and remained there for 15 s, the same period as was allowed for the successful animals. 24 h after the last navigation training trial, the platform was removed, and each rat was subjected to the probe trial, during which the rats were placed into the tank at the midpoint of the edge of quadrant W and allowed to swim in the tank for 60 s. The time spent in the target quadrant (quadrant E) and the time of crossing the location where the platform had been fixed were recorded as a measure of spatial memory. All measurements were recorded by an AHD camera 2‐MP, Model No: cp‐B20M3, Lens: 3.6 mm‐3MP (HIKVISION, Hangzhou, China) (Figure 2).

### 2.7. Tissue Preparation

At the end of the experiments following the intraperitoneal injection of ketamine (80 mg/kg, i.p.) and xylazine (10 mg/kg, i.p.), the animals were transcardially perfused with cold normal saline followed by 4% paraformaldehyde in 0.1 M PBS with a pH of 7.4. Immediately after perfusion, the brain was dissected and immersed in 4% paraformaldehyde for 24 h at 4°C.

After perfusion and fixation, brain tissues were processed, embedded in paraffin, and coronally sectioned at a thickness of 8 μm using a microtome (include microtome model if possible). Sections were mounted on gelatin‐subbed glass slides to prevent detachment. Sections were deparaffinized in xylene (2 changes, 10 min each). They were then rehydrated through a graded series of ethanol solutions (100%, 95%, 70%—5 min each) and finally rinsed in distilled water. Slides were stained in a 0.5% Cresyl Violet (Nissl) solution (prepared in distilled water) for 10–15 min at 40°C–50°C. After staining, slides were briefly rinsed in distilled water to remove excess stain. Differentiation was performed in 95% ethanol (checked microscopically to achieve optimal contrast between Nissl bodies and the background). Slides were dehydrated through a graded series of alcohols (95% and 100%—2 changes, 2 min each), cleared in xylene (2 changes, 5 min each), and coverslipped using a permanent mounting medium (DPX).

### 2.8. Assessment of Dark Neurons and Thickness

Nissl‐stained sections were examined under a light microscope (Olympus BX53 at 400× magnification) by an experimenter blinded to the group assignments. Dark neurons were identified based on well‐established morphological criteria distinguishing them from healthy neurons and glial cells [14]. The definitive criteria for a dark neuron included (a) cellular shrinkage: a markedly reduced and often triangulated or pyknotic somatic profile, (b) hyperbasophilia: intense, homogeneous dark blue‐purple staining of the cytoplasm and nucleus with a loss of normal Nissl substance pattern, (c): dendritic alterations: the presence of corkscrew‐like, beaded, or fragmented proximal dendrites. Healthy, viable neurons were identified by their large, spherical, and lightly basophilic somata, distinct nucleoli, and clearly visible Nissl substance. Glial cells were distinguished by their significantly smaller size, darker and more condensed nuclei, and lack of visible cytoplasm or Nissl substance under the used magnification. Dark neurons were identified based on described well‐established morphological criteria distinguishing them from healthy neurons and glial cells.

For each animal, three coronal hippocampal sections were consistently obtained between Bregma −2.8 mm and −3.8 mm (dorsal hippocampus), as defined by the Paxinos and Watson rat brain atlas. All animals were sectioned at these standardized levels to ensure anatomical comparability across groups. Within the CA1 and DG regions, dark neurons were counted in a predefined area of 0.1 mm^2^ per region using a counting grid within the microscope eyepiece. The thickness of the CA1 and DG layers was measured at three standardized points per region per section using the calibration tool of the microscope’s imaging software. Data are expressed as the percentage of dark neurons relative to the total number of neurons in the counted area.

The thickness of the hippocampal CA1 and DG regions was measured on the same Nissl‐stained sections used for dark neuron quantification. For the CA1 region, thickness was measured as the vertical distance spanning the entire pyramidal cell layer, from the stratum oriens to the stratum radiatum. For the DG, thickness was measured as the vertical distance of the granular cell layer at the crest of the superior blade. For each animal, three measurements were taken at standardized locations within each region per section (at the medial, central, and lateral aspects of the DG blade and the CA1 layer). All measurements were performed using the calibrated measurement tool in the microscope’s proprietary software at a 100× or 200× magnification. The final thickness value for each region per animal was calculated as the average of all measurements taken across the analyzed sections.

### 2.9. Immunohistochemistry

To assess the expression of GFAP, an immunohistochemical analysis was conducted. Brain sections were deparaffinized and incubated with 5% normal goat serum (NGS) and 1% bovine serum albumin (BSA) in PBS for 60 min. Rabbit anti‐GFAP was then applied overnight at 4°C. Subsequently, goat antirabbit IgG (FITC) was used for 60 min at room temperature and the nuclei were stained with DAPI. Immunoreactive cells were analyzed using a fluorescent microscope, and the percentage of immunopositive cells per area was calculated using the formula: (number of positive cells × 100)/(the total number of nuclei) formula.

### 2.10. Statistical Analyses

The Shapiro–Wilk test was used to assess the normality, and Levene’s test was conducted to check for the homogeneity of variances. Based on the normality and homogeneity of data, one‐way ANOVA was used to compare variables among the study groups. For post hoc pairwise comparisons between groups, the least significant difference (LSD) test was used. The significance level was set at *p* < 0.05. All statistical analyses were conducted using SPSS® 23.0 (IBM Corporation, Armonk, NY, USA) for Windows®.

## 3. Results

### 3.1. Body Weight

Regarding the weight of the animals, weights were compared before and after treatment. It was found that there was no significant change in weight posttreatment compared to pretreatment in the CON (230.33 ± 25.28 vs. 233.67 ± 24.44 gr, *p* = 0.341) and CS + R (284.36 ± 2.32 vs. 279.83 ± 2.31 gr, *p* = 0.342) groups. However, in the CS + ST group (229.50 ± 17.76 vs. 232.03 ± 26.67, *p* = 0.003), CS group (229.50 ± 17.76 vs. 215.95 ± 12.42, *p* = 0.003), and in ST group (256.58 ± 13.54 vs. 224.17 ± 7.46 gr, *p* = 0.001), the weight significantly decreased in posttreatment compared to pretreatment.

### 3.2. GFAP in CA1 Region

The study found significant differences in GFAP (%) in the CA1 region across five groups (*F* (4, 25) = 11.60, *p* < 0.001). Post hoc analyses revealed that the GFAP level was highest in the CS group (15.83 ± 1.80) compared to the ST, CON, CS + recovery, and CS + ST groups (*p* = 0.002 vs. CON (9.43 ± 3.18)). Conversely, GFAP levels were lowest in the ST group (4.38 ± 0.52) compared to those in all other groups (*p* < 0.01 vs. CON). Additionally, GFAP was higher in the CS (*p* < 0.001) and CS + recovery (11.89 ± 4.38; *p* = 0.02) groups compared to that in the CS + ST group (7.41 ± 4.02). The ST (*p* < 0.001) and CS + recovery (*p* = 0.03) groups had lower GFAP levels compared to the CS group. Lastly, the GFAP level in the CS + recovery group was significantly higher than in the ST group (*p* < 0.001) (Figure 3).

Figure 3Glial fibrillary acidic protein (GFAP) immunoreactivity in the hippocampal CA1 and DG regions (%). (a) Representative micrographs of the CA1 region. (b) Representative micrographs of the DG region. Representative fluorescent images of hippocampal CA1 and DG regions obtained from coronal sections between Bregma −2.8 mm and −3.8 mm. (c, d) Quantitative analysis of GFAP‐positive cells. Data are expressed as the mean ± S.E.M. ^∗^ *p* < 0.001, significantly different from the control group. ^∗∗^ *p* < 0.001, significantly different from the CS + ST group. ^∗∗∗^ *p* < 0.001, significantly different from the CS group. ^#^ *p* < 0.001, significantly different from the ST group. ^##^ *p* < 0.001, significantly different from the CS + recovery group. CON = control, CS = chronic mild stress, CS + recovery = chronic mild stress followed by recovery time, CS + ST = chronic mild stress followed by swimming training, ST = swimming training. The sample size for quantitative analysis was *n* = 6 per group.

**Figure:**
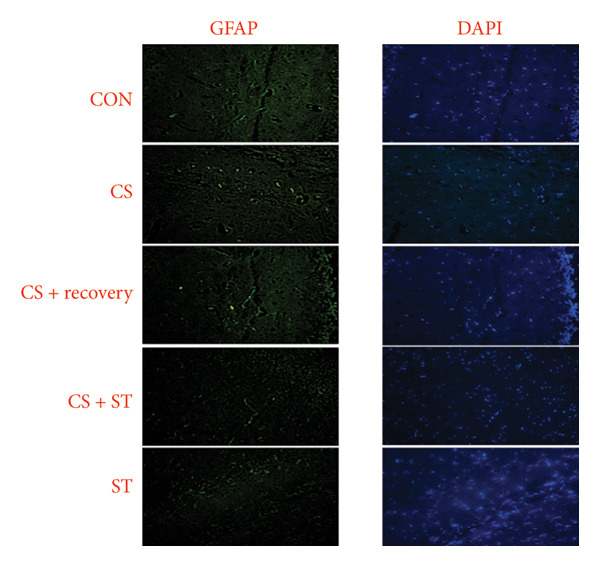
(a)

**Figure:**
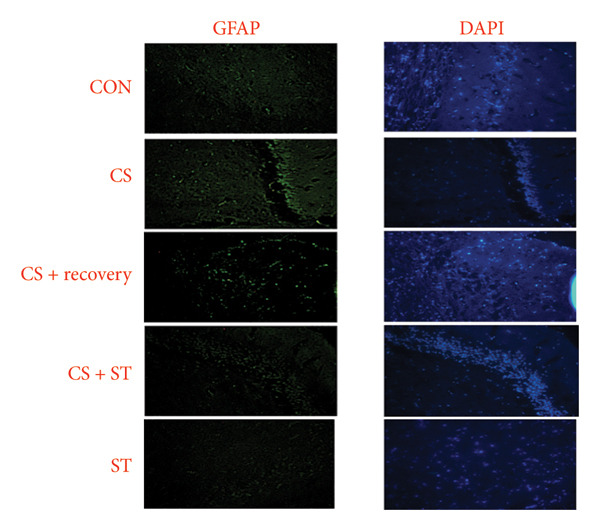
(b)

**Figure:**
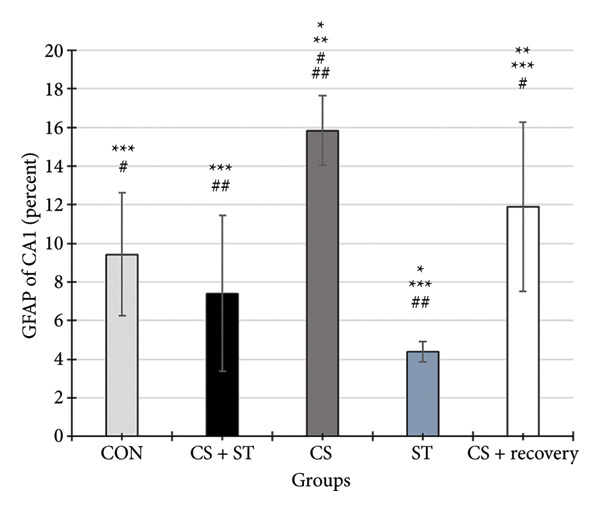
(c)

**Figure:**
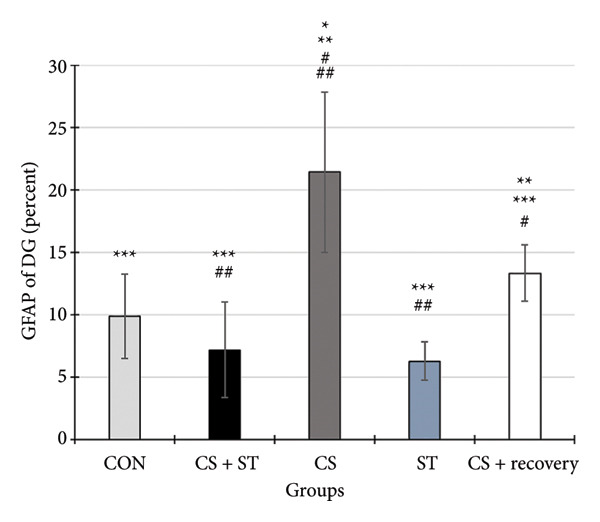
(d)

### 3.3. GFAP in DG Region

GFAP (%) in the DG region showed significant differences among the five groups (*F* (4, 25) = 14.98, *p* < 0.001). Post hoc analyses revealed that the CS group had the highest GFAP levels (21.42 ± 6.43) compared to the ST, CON, CS + recovery, and CS + ST groups (*p* < 0.001 vs. CON (9.90 ± 3.38)). Conversely, the ST group had the lowest GFAP levels (6.29 ± 1.54) compared to all other groups (*p* < 0.001 vs. CS). The CS group also had significantly higher GFAP levels than both the CS + ST group (7.19 ± 3.83; *p* < 0.001) and the CS + recovery group (13.34 ± 2.25; *p* = 0.01). The CS + recovery group had significantly lower GFAP levels compared to the CS group (*p* < 0.001) but higher levels than the ST group (*p* = 0.004) (Figure 3).

### 3.4. Dark Neurons in CA1 Region

Dark neuron (%) in the CA1 region showed significant differences across the five groups (*F* (4, 25) = 68.92, *p* < 0.001). Post hoc analyses indicated that dark neuron levels were highest in the CS group (51.55 ± 6.62) and lowest in the ST group (9.51 ± 0.82) compared to those in all other groups (*p* < 0.0001 vs. CON (20.55 ± 4.33)). Levels were also significantly higher in the CS + recovery group (30.07 ± 6.69; *p* < 0.001 vs. CON) compared to that in the control. Furthermore, when compared to the CS + ST group (20.24 ± 2.37), dark neuron levels were significantly higher in the CS and CS + recovery groups and significantly lower in the ST group. Compared to the CS group, dark neuron levels were significantly lower in both the ST and CS + recovery groups (*p* < 0.001). Finally, the CS + recovery group had significantly higher levels of dark neurons than the ST group (*p* < 0.001) (Figure 4).

Figure 4Dark cell of CA1 and DG (%). (a) Representative micrographs of the CA1 region. (b) Representative micrographs of the DG region. Representative Nissl‐stained images of the CA1 pyramidal cell layer and DG granular cell layer (Bregma −2.8 mm to −3.8 mm). Dark neurons were quantified from standardized 0.1‐mm^2^ fields of view across all groups. (c, d) Quantitative analysis of GFAP‐positive cells. Data are expressed as the mean ± S.E.M. ^∗^ *p* < 0.001, significantly different from the control group. ^∗∗^ *p* < 0.001, significantly different from the CS + ST group. ^∗∗∗^ *p* < 0.001, significantly different from the CS group. ^#^ *p* < 0.001, significantly different from the ST group. ^##^ *p* < 0.001, significantly different from the CS + recovery group. CON = control, CS = chronic mild stress, CS + recovery = chronic mild stress followed by recovery time, CS + ST = chronic mild stress followed by swimming training, ST = swimming training. The sample size for quantitative analysis was *n* = 6 per group.

**Figure:**
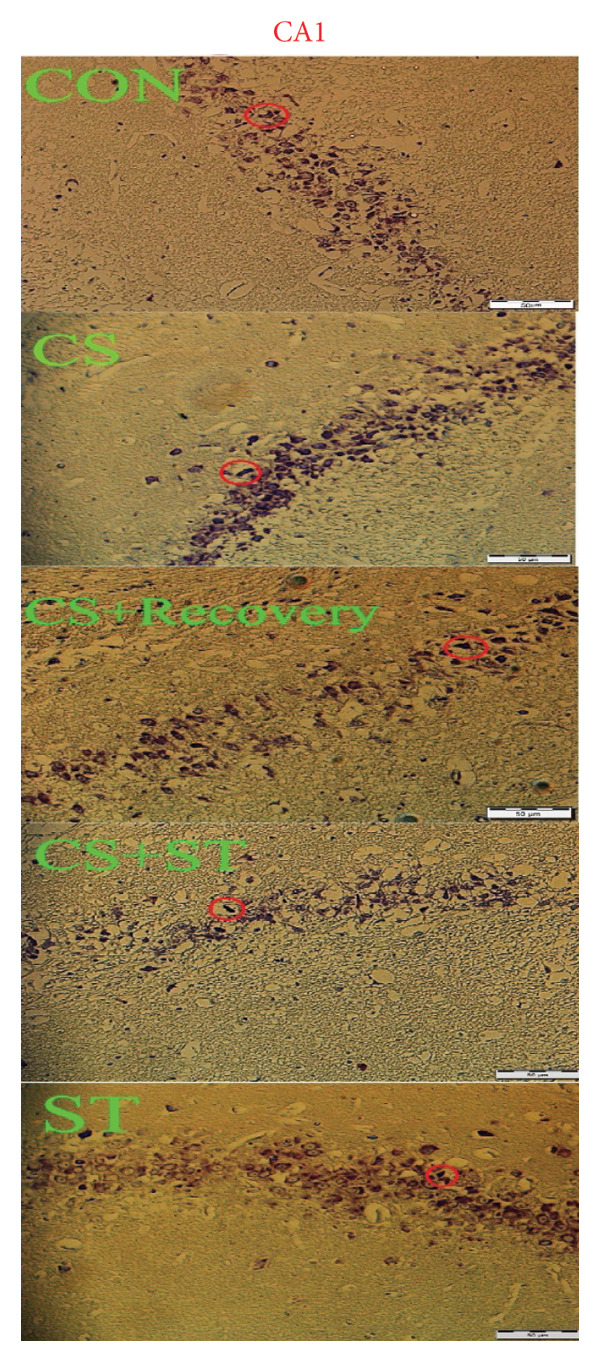
(a)

**Figure:**
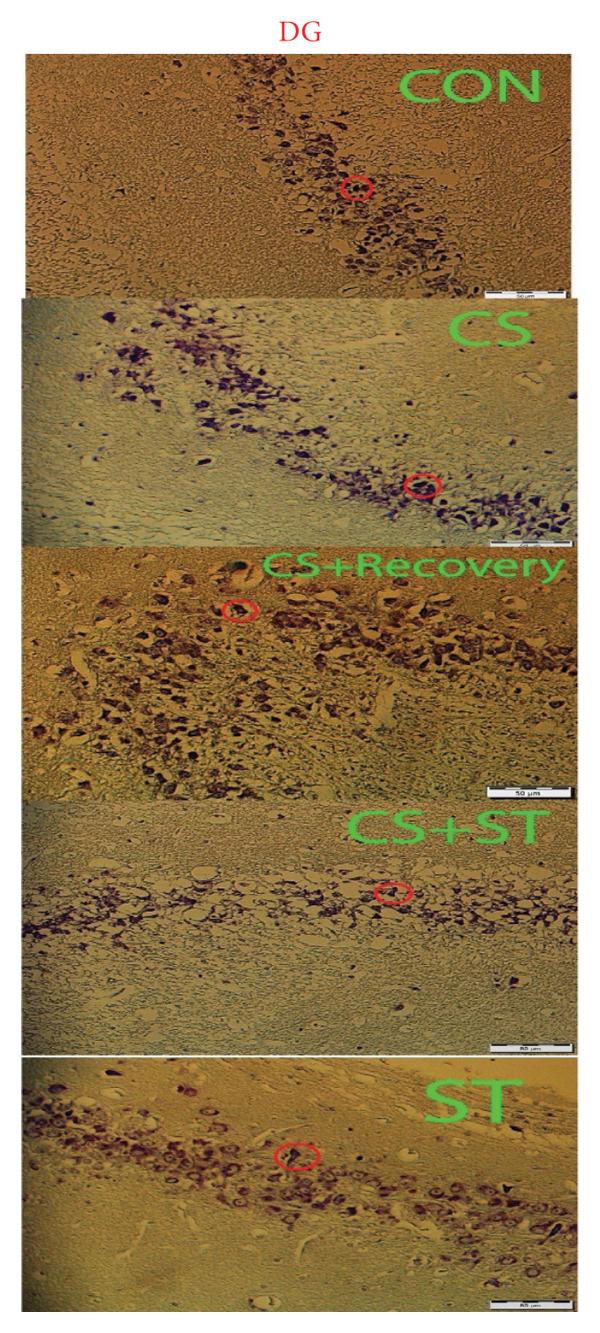
(b)

**Figure:**
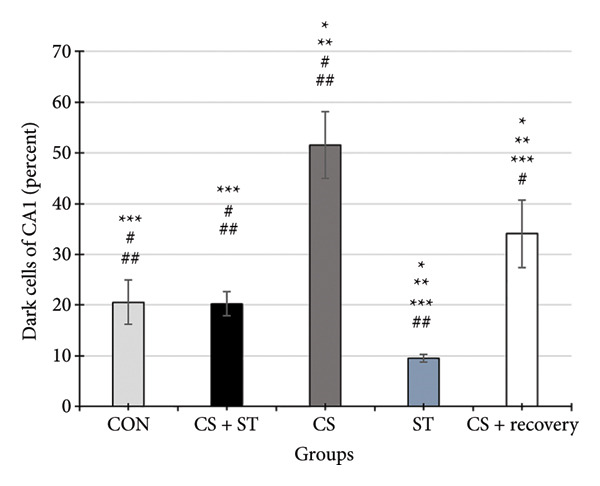
(c)

**Figure:**
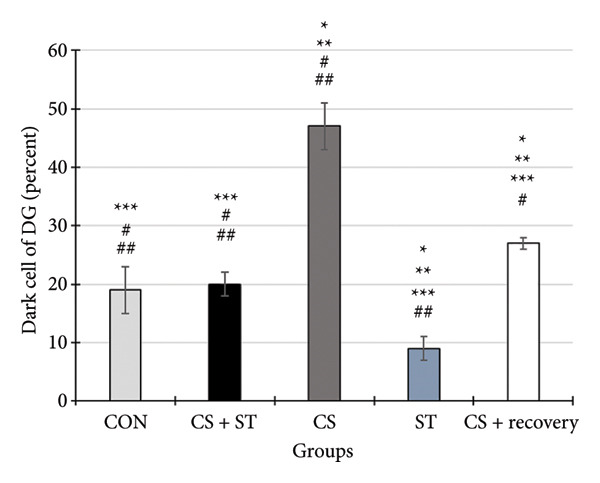
(d)

### 3.5. Dark Neurons in DG Region

Dark neurons (%) in the DG region were significantly different across the five group conditions (*F* (4, 25) = 128.13, *p* < 0.001). Post hoc analyses revealed that dark neuron levels were highest in the CS group (47.71 ± 4.14) and lowest in the ST group (9.21 ± 2.06) compared to those in all other groups (*p* < 0.001 vs. CON (19.15 ± 4.34)). Levels were also significantly higher in the CS + recovery group (27.4 ± 1.56; *p* < 0.0001 vs. CON). When compared to the CS + ST group (20.64 ± 2.64), dark neuron levels were significantly higher in the CS and CS + recovery groups and significantly lower in the ST group. Additionally, compared to the CS group, dark neuron levels were significantly lower in both the ST and CS + recovery groups (*p* < 0.001). Finally, the CS + recovery group had significantly higher dark neuron levels than the ST group (*p* < 0.001).

### 3.6. Thickness of CA1 Region

The thickness of the CA1 region (μm) showed significant differences across the five groups (*F* (4, 25) = 382.51, *p* < 0.001). Post hoc analyses revealed that CA1 thickness was highest in the ST group (92.19 ± 4.82) and lowest in the CS group (30.75 ± 1.38) compared to all other groups (*p* < 0.001 vs. CON (70.14 ± 1.46)). Compared to the control, thickness was also significantly lower in the CS + recovery group (53.20 ± 3.09; *p* < 0.001). When compared to the CS + ST group (72.35 ± 2.18), the thickness of CA1 was significantly lower in both the CS and CS + recovery groups but significantly higher in the ST group. CA1 thickness was significantly greater in both the ST and CS + recovery groups compared to the CS group (*p* < 0.001). In contrast, thickness in the CS + recovery group was significantly lower than in the ST group (*p* < 0.001).

### 3.7. Thickness of DG Region

The thickness of the DG region (μm) differed significantly among the five groups (*F* (4, 25) = 429.58, *p* < 0.001). Post hoc analyses indicated that DG thickness was highest in the ST group (93.78 ± 3.87) and lowest in the CS group (34.04 ± 1.76) compared to that in all other groups (*p* < 0.001 vs. CON (68.78 ± 1.21)). Compared to the control, thickness was also significantly lower in the CS + recovery group (52.15 ± 3.30; *p* < 0.0001). When compared to the CS + ST group (71.69 ± 2.12), DG thickness was significantly lower in both the CS and CS + recovery groups and significantly higher in the ST group. Additionally, DG thickness was significantly greater in both the ST and CS + recovery groups when compared to that in the CS group (*p* < 0.001). In contrast, the thickness of DG was significantly lower in the CS + recovery group compared to that in the ST group (*p* < 0.001) (see Figure 5).

Figure 5Thickness of CA1 and DG (%). (a) Representative micrographs of the CA1 region. (b) Representative micrographs of the DG region. Representative images showing the pyramidal layer (CA1) and granular layer (DG) at Bregma −2.8 mm to −3.8 mm. (c, d) Quantitative analysis of GFAP‐positive cells. Data are expressed as the mean ± S.E.M. ^∗^ *p* < 0.001, significantly different from the control group. ^∗∗^ *p* < 0.001, significantly different from the CS + ST group. ^∗∗∗^ *p* < 0.001, significantly different from the CS group. ^#^ *p* < 0.001, significantly different from the ST group. ^##^ *p* < 0.001, significantly different from the CS + recovery group. CON = control, CS = chronic mild stress, CS + recovery = chronic mild stress followed by recovery time, CS + ST = chronic mild stress followed by swimming training, ST = swimming training. The sample size for quantitative analysis was *n* = 6 per group.

**Figure:**
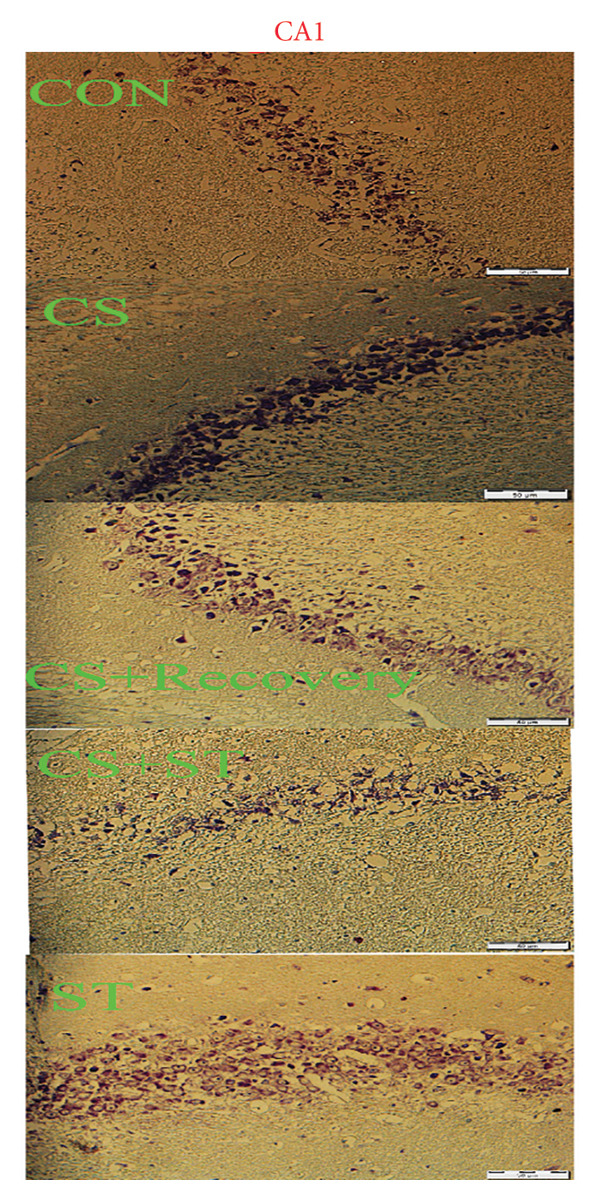
(a)

**Figure:**
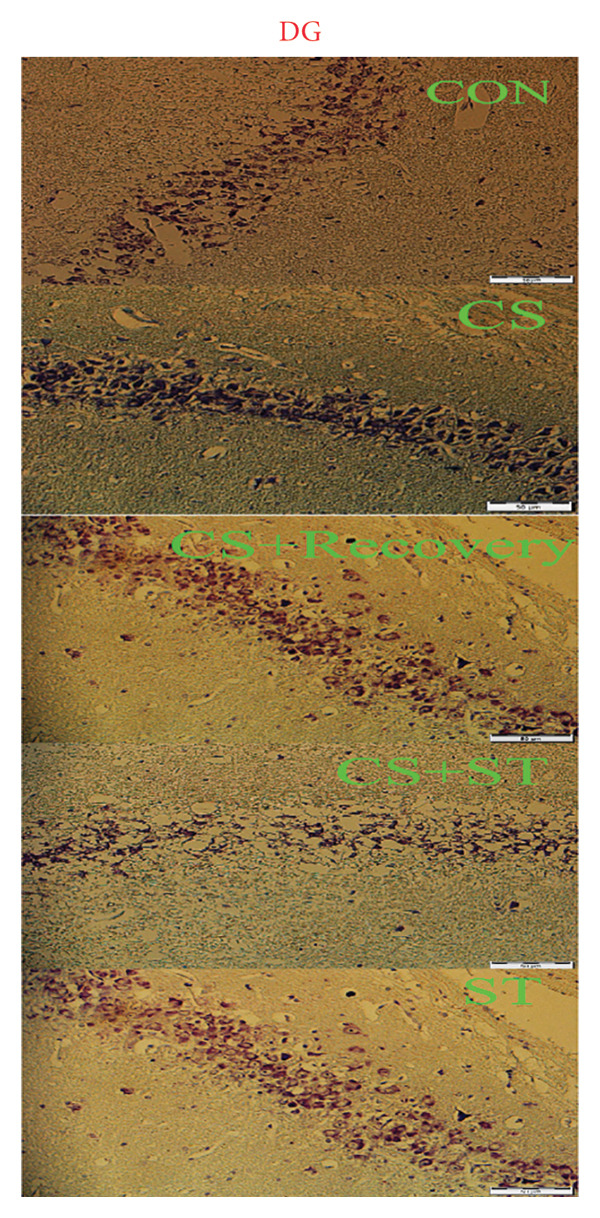
(b)

**Figure:**
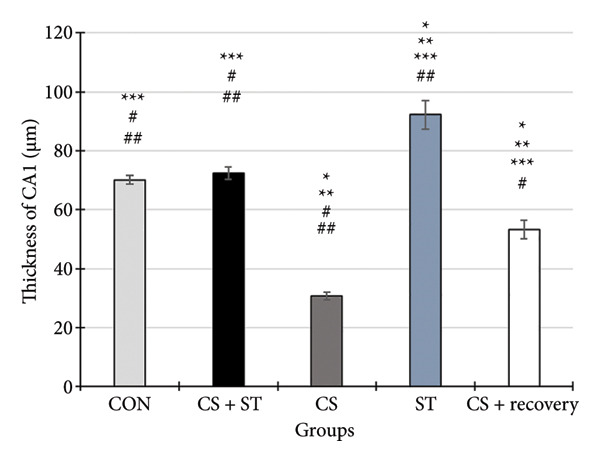
(c)

**Figure:**
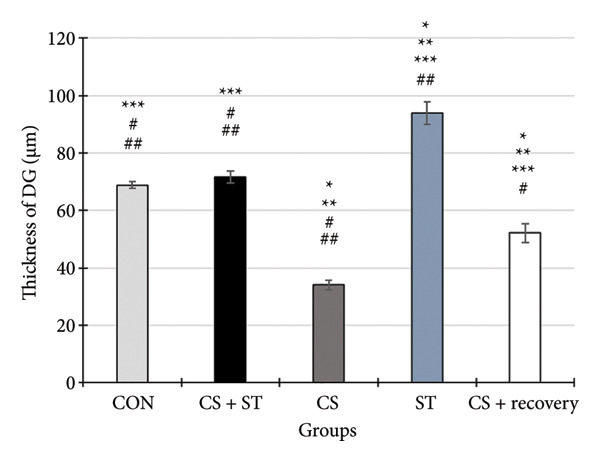
(d)

### 3.8. Percentage of Time Spent in the Target Quarter (PSTQ) in MWM

**Figure 6:**
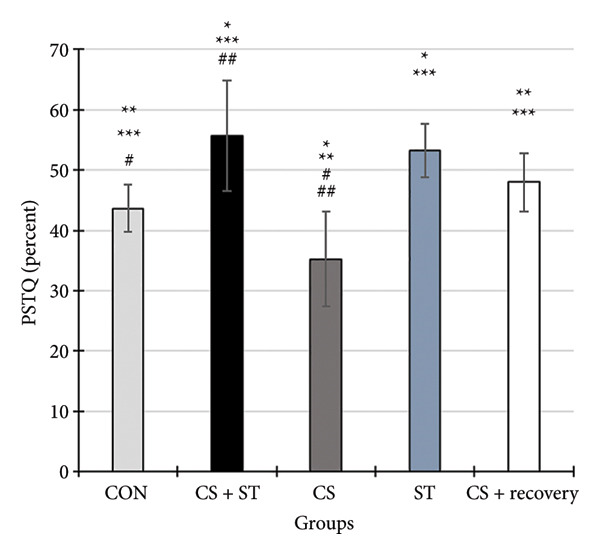
Percentage of the time spent in the target quarter (PSTQ) (%). Data are expressed as the mean ± S.E.M. ^∗^ *p* < 0.001 significantly different from the control group. ^∗∗^ *p* < 0.001, significantly different from the CS + ST group. ^∗∗∗^ *p* < 0.001, significantly different from the CS group. ^#^ *p* < 0.001, significantly different from the ST group. ^##^ *p* < 0.001, significantly different from the CS + recovery group. CON = control, CS = chronic mild stress, CS + recovery = chronic mild stress followed by recovery time, CS + ST = chronic mild stress followed by swimming training, ST = swimming training.

**Figure 7:**
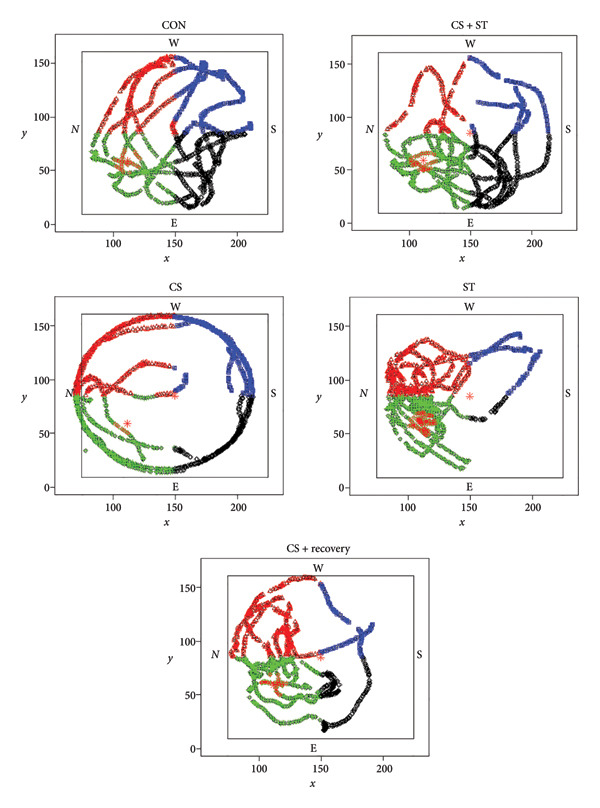
Schematic images of Morris water maze testing. CON = control, CS = chronic mild stress, CS + recovery = chronic mild stress followed by recovery time, CS + ST = chronic mild stress followed by swimming training, ST = swimming training.

The PSTQ scores differed significantly among the five group conditions (*F* (4, 25) = 9.63, *p* < 0.001). Post hoc analyses revealed that PSTQ scores were highest in the CS + ST group (55.63 ± 9.16) and lowest in the CS group (35.23 ± 7.89) compared to all other groups (*p* = 0.003 and *p* = 0.032, respectively, vs. CON (43.62 ± 3.91)). Scores were also significantly higher in the ST group (53.19 ± 4.46; *p* = 0.016 vs. CON). When compared to the CS + ST condition, the PSTQ was significantly lower in both the CS (*p* < 0.001) and CS + recovery (47.97 ± 4.85; *p* = 0.049) groups. Additionally, compared to the CS group, the PSTQ was significantly higher in both the ST (*p* < 0.001) and CS + recovery (*p* = 0.016) groups (Figures 6 and 7).

### 3.9. The First Time to Reach the Platform (FTRP) in MWM

**Figure 8:**
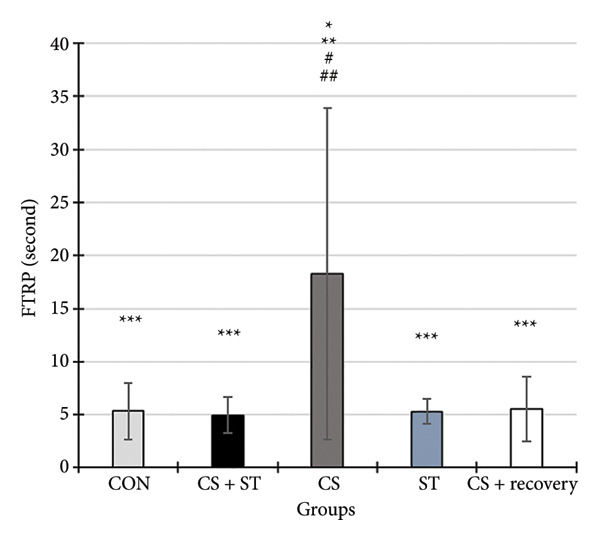
The first time to reach the platform (FTRP) (second). Data are expressed as the mean ± S.E.M. ^∗^ *p* < 0.001 significantly different from the control group. ^∗∗^ *p* < 0.001, significantly different from the CS + ST group. ^∗∗∗^ *p* < 0.001, significantly different from the CS group. ^#^ *p* < 0.001, significantly different from the ST group. ^##^ *p* < 0.001, significantly different from the CS + recovery group. CON = control, CS = chronic mild stress, CS + recovery = chronic mild stress followed by recovery time, CS + ST = chronic mild stress followed by swimming training, ST = swimming training.

The FTRP differed significantly among the five groups (*F* (4, 25) = 3.81, *p* = 0.015). Post hoc analyses revealed that the FTRP was highest in the CS group (18.25 ± 15.63) and lowest in the CS + ST group (4.96 ± 1.67) compared to all other groups (*p* = 0.005 and *p* = 0.004, respectively, vs. CON (5.31 ± 2.70)). Compared to the CS group, the FTRP was significantly lower in both the ST (5.28 ± 1.19; *p* < 0.005) and CS + recovery (5.22 ± 3.06; *p* = 0.006) groups (Figures 7 and 8).

## 4. Discussion

The purpose of the present study was to evaluate the effect of CS and swimming training on hippocampal morphology and spatial memory. Our principal findings demonstrate that swimming training effectively reversed CS‐induced deficits. Specifically, swimming reduced astrogliosis (GFAP) and the number of dark neurons—direct indicators of neuronal injury—while increasing the thickness of the CA1 and DG regions. These morphological improvements were paralleled by enhanced performance in the MWM. A recovery period without intervention provided partial benefits, but these were consistently surpassed by the active swimming intervention.

### 4.1. Morphological Change in Hippocampus: GFAP, Dark Neurons, and Thickness of Hippocampus

This study demonstrated that swimming training effectively mitigates the detrimental morphological effects of CMS on the hippocampus in young male rats. Specifically, swimming reduced astrogliosis—as indicated by decreased GFAP expression—and lowered the percentage of dark neurons in both the CA1 and DG regions. Swimming also increased the thickness of these hippocampal subfields, suggesting reduced neuronal damage and enhanced structural integrity. A period of recovery time without any treatment after CS (in the CS + recovery group) could only reduce GFAP and dark neurons and increase thickness and special memory compared to the CS condition.

The reduction in reactive astrocytes (GFAP‐positive) highlights the probable attenuation of neuroinflammatory processes commonly induced by CS. Preclinical studies have demonstrated that CS causes dendritic atrophy and decreased spine density in pyramidal neurons of the hippocampus and the medial prefrontal cortex (mPFC) [38, 39]. CS simultaneously leads to shortening of branches and a decrease in the number of astrocytes in rodents, while exercise can lead to a significant increase in astrocyte projections (number of bifurcation points and area covered by these projections) [40]. In addition to the increase in astrocytic projections due to exercise activity, it seems that astrocytic projections are more directed to the DG region of the hippocampus, the region of the hippocampus that has the highest rate of BDNF synthesis and release after exercise activity [41]. While previous research [25, 42] has linked exercise broadly to astrocyte remodeling and neurotrophic factor modulation, our findings confirm these effects in the context of swimming, which uniquely involves whole‐body engagement and aquatic environmental factors. These features may contribute distinct neuroprotective pathways, such as specific modulation of corticosterone levels and oxidative stress, beyond what is observed in land‐based exercises.

Gokdemir et al. reported that exercise in multiple sessions may increase the number of cells in the hippocampus, prefrontal cortex, and amygdala [25]. Also, people who performed an exercise program for about 6 weeks had a more stable brain structure than those who did not exercise [42]. While in people who did not do exercise, the hippocampal volume and BDNF were reduced [43]. It seems that the beneficial effects of exercise on the hippocampus are due to improved neurogenesis [25, 44], maturation of newborn nerve cells, increased dendritic complexity, increased astrocyte ramification and the length of their processes, a structural arrangement close to neural activity [27, 28, 41], and increased length of dendritic spines [45, 46].

The main goal for exercise in the CNS is the hippocampal region, especially the DG region [47]. Dendritic spine density [48] and the rate of neurogenesis increase in this area as a result of exercise [47]. Although the exact mechanism of these morphological effects needs to be clarified, it seems that the most important mechanism in this process is to increase the synthesis and availability of neurotrophins, especially BDNF [49]. In general, the effects of exercise on astrocytes, which include morphological changes, are associated with an increase or suppression in the level of GFAP in animals [27–29, 50].

Another interesting finding of this study showed that swimming training following CS reduced GFAP levels and dark neurons while increasing the thickness of the CA1 and DG regions of the hippocampus compared to the control group. A study showed that 4 weeks of training increased the volume of the CA and DG in the CMS/training group compared with those in the CMS/standard group [51]. However, the exact cellular mechanism of these changes has not been clarified yet. CS increases astrogliosis [52, 53], while exercise increases the size of astrocytes [28] and can also reduce astrogliosis [28] and reactive astrocytes [29] by expressing GFAP. The difference in GFAP levels can be attributed to astrocyte heterogeneity as well as differences in the type of exercise intervention. In addition, factors such as daily access period, exercise duration, and age of start of intervention vary significantly between studies [54]. In response to CNS damage, astrocytes can be transferred to a reactive state, and this astrocyte reaction is associated with altered expression of many genes, increased GFAP expression, and functional and morphological changes [55, 56]. Another possible mechanism of exercise‐induced changes in the morphology of the hippocampus following CS may be related to corticosterone. As such, one of our previous research findings revealed that CS increased corticosterone levels in rats, while swimming training decreased the corticosterone levels compared to CS conditions [57].

The recovery group (CS + recovery) showed partial spontaneous morphological improvements compared to stressed rats without exercise intervention, indicating that some resolution of neuroinflammation and restoration of hippocampal structure can occur naturally over time. However, these changes were less robust relative to active swimming, underscoring the enhanced neuroprotective advantage conferred by exercise interventions.

### 4.2. Spatial Memory

Accompanying the morphological improvements, swimming training significantly enhanced spatial memory performance. Marks of increased spatial memory included decreased latency to find the hidden platform and increased time spent in the target quadrant in the MWM. CMS impaired spatial memory, consistent with the previous literature linking stress to neuroinflammation, disrupted LTP, and decreased neurogenesis in hippocampal circuits critical for memory [58]. Swimming training after stress exposure improved these deficits, though with somewhat smaller effects than in nonstressed trained rats, suggesting partial recovery abilities alongside exercise‐induced enhancement.

The most important area for separating spatial memory patterns in the hippocampus is the DG area. Damage to this area causes a disturbance in spatial memory [10, 59]. In the DG of the hippocampus, new nerve cells are born in mammals, a process called adult neurogenesis [60, 61], which plays a key role in pattern separation in spatial memory [62]. The present study found that CS reduces spatial memory. Reduction of spatial memory may be due to some reasons. An increase in the expression of proinflammatory cytokines in the hippocampus such as TNF, IL‐1B, and IL6 plays an important role in memory and learning [63, 64]. Inflammatory cytokines can directly affect neural functions, LTP, the secretion of glutamate, glutamate receptor density, and cell signaling pathways associated with learning and memory [65]. Also, chronic exposure to stress disrupts various stages of hippocampal‐dependent memory and also causes structural and functional changes in the hippocampus [66]. For example, CS decreases neurogenesis in the DG and increases atrophy of apical dendrites in the CA3 region of the hippocampus [38].

Limited studies have been conducted focusing on the effect of physical activity and exercise on the hippocampus and spatial memory patterns in humans. A study indicated that physical activity improved object recognition in young adults by altering the functional connection between the hippocampal region (DG and CA regions) and the cortical regions involved in recall, suggesting that physical activity can affect functional changes of the hippocampus without structural change in adults [67]. However, the findings of the present study indicated that an increase in spatial memory occurred simultaneously with an increase in the thickness and a decrease in the number of dark neurons and astrogliosis in different areas of the hippocampus.

Exercise and physical activity can increase memory in the hippocampus by network wiring [10]. In addition, exercise improves synaptic flexibility in adult‐born neurons. Voluntary exercise increases short‐term synaptic flexibility from the lateral entorhinal cortex to adult‐born neurons [68]. Exercise also increases innervation of the entorhinal cortex and areas important for spatial memory and the production of theta rhythms, including adult‐born neurons, such as the caudomedial entorhinal cortex, middle septum, and upper internal mammalian nuclei [68]. Exercise can also modify the expression of genes necessary for synaptic transmission in the lateral entorhinal cortex [69]. Probably, swimming training could improve memory through some observed structural changes in the hippocampus through possible mentioned mechanisms.

Another notable finding of the present study was that swimming training following CS increased spatial memory compared to the control group, but these increases were less than those of the swimming group and were similar to the control group in some indices. Meanwhile, swimming training following CMS could increase spatial memory compared to CON, CS, and CS + recovery. Jahangiri et al. indicated that moderate training improved memory impairments by attenuating the hippocampal cytokine levels and brain oxidative damage [22]. Numerous studies have reported that regular exercise can have an anti‐inflammatory effect and modify the increase in levels of inflammatory cytokines [70, 71]. Some other cellular and molecular mechanisms have been suggested for this change due to aerobic exercise, including increased expression of markers of synaptic flexibility, such as synaptophysin (SYP) and postsynaptic 95‐density protein‐95 (PSD‐95) and growth factors in the hippocampus [72]. Swimming exercise ameliorates mood disorder and memory impairment by enhancing neurogenesis and serotonin expression and inhibiting apoptosis in social isolation [24].

While many synaptic plasticity mechanisms—such as increased expression of SYP, PSD‐95, and neurotrophic factors like BDNF—are well documented in treadmill and wheel running models, future research should clarify their direct involvement in swimming. Swimming’s unique physiological demands may also affect neurocircuitry and neurochemical signaling differently, potentially involving distinct respiratory and cardiovascular adaptations.

The partial recovery observed in the CS + recovery group may reflect endogenous neurorestorative processes, such as gradual normalization of the HPA axis hyperactivity, reduction in proinflammatory cytokines, and spontaneous synaptic reorganization [58, 66]. CS is known to elevate glucocorticoids and inflammatory mediators, which impair neurogenesis and synaptic plasticity. Over time, removal of stressors may allow homeostatic mechanisms to restore baseline function, albeit slowly and incompletely. This aligns with clinical observations that while some individuals recover from stress exposure without intervention, others develop persistent cognitive deficits, highlighting the value of active therapeutic strategies. This underscores the therapeutic advantage of structured swimming training over passive recovery. This suggests that swimming does not merely permit recovery but actively enhances neuroplasticity, possibly through exercise‐induced neurotrophic support and anti‐inflammatory effects. These findings support the concept that structured physical activity is a potent enhancer of brain resilience, beyond what can be achieved through rest alone.

### 4.3. Special Mechanisms Related to Swimming

While numerous studies have demonstrated that land‐based aerobic exercise (e.g., treadmill running, voluntary wheel running) enhances hippocampal neurogenesis [19, 68], synaptic plasticity [72], and BDNF expression [49], the neurobiological mechanisms underlying the benefits of swimming as a distinct exercise modality are less extensively characterized. In the present study, we observed significant improvements in hippocampal morphology and spatial memory following swimming training. However, it is important to distinguish between mechanisms directly demonstrated in swimming models and those reasonably extrapolated from other forms of exercise. Several mechanisms are directly supported by evidence from swimming studies. For instance, swimming has been consistently shown to reduce stress‐induced elevations in corticosterone levels in rodents [24, 57], which aligns with our findings of stress mitigation and supports the role of swimming in modulating the HPA axis. Additionally, swimming promotes cell proliferation in the DG of rats [24, 33], indicating enhanced hippocampal neurogenesis, a finding that corroborates our observed structural improvements in the DG and CA1 regions. Furthermore, studies such as that by Park et al. have demonstrated that swimming in adolescent rats reduces hippocampal apoptosis and increases serotonin expression [24], paralleling our results of reduced dark neuron counts and neuronal damage. Behavioral outcomes are also consistent with prior work, as several studies using swimming as an intervention report improved performance in the MWM [24, 25], reinforcing the validity of our spatial memory findings.

In contrast, some commonly cited mechanisms, while plausible, are primarily derived from research on land‐based exercise and remain extrapolations in the context of swimming. For example, upregulation of BDNF, SYP, and PSD‐95 is well documented following treadmill or wheel running [72], but direct evidence from swimming paradigms is limited. Since our study did not measure these molecular markers, their involvement in swimming‐induced neuroprotection remains hypothetical and should be investigated in future studies. Similarly, enhancements in LTP and modulation of hippocampal theta rhythms have been primarily observed in voluntary wheel‐running models [69, 73] and have not been directly assessed in swimming protocols. Therefore, claims that swimming improves memory through such network‐level mechanisms should be regarded as speculative at this stage. Importantly, swimming presents unique physiological challenges compared to terrestrial exercise, including whole‐body immersion, respiratory adaptation, hydrostatic pressure, and thermoregulatory demands, which may differentially influence the central nervous system function. The rhythmic breathing and immersion associated with swimming may enhance parasympathetic tone, potentially leading to greater suppression of neuroinflammation [57]. The reduced gravitational load during swimming may also decrease musculoskeletal stress while maintaining cardiovascular intensity, possibly allowing for longer or more sustainable training sessions, particularly under conditions of prior stress exposure. Moreover, some evidence suggests that swimming may lead to greater activation of brainstem and cerebellar regions [74], which could indirectly modulate hippocampal function through neuromodulatory pathways such as the locus coeruleus–norepinephrine system. Thus, although swimming shares many neuroprotective outcomes with other aerobic exercises, its distinct physiological context may engage partially unique neurobiological pathways, highlighting the need for further comparative research to elucidate the specific mechanisms through which swimming exerts its beneficial effects on the brain structure and function.

## 5. Strength and Limitations

One of the novel strengths of this study was the simultaneous evaluation of the effects of swimming on hippocampal morphology and spatial memory under both stressed and nonstressed conditions, as well as the comparison of recovery time without any intervention. Additionally, this study uniquely focused on swimming as the mode of exercise, demonstrating its positive effects on hippocampal morphology and spatial memory, which are partly consistent with findings from previous studies on land‐based exercises. This suggests that swimming could be a valuable approach for future research in this area.

A limitation of this study was the lack of direct measurement of BDNF and other neurotrophic factors, which would have solidified the mechanistic link between swimming and the observed neuroprotection. Future studies should directly quantify these molecular pathways in this model. Furthermore, a direct comparison of swimming with land‐based exercise could reveal unique mechanisms specific to the aquatic environment. While images were captured from a standardized field of view within the dorsal hippocampus for quantification, the representative selection shown here may not originate from perfectly matched anatomical levels, a limitation of the study.

## 6. Conclusion

In conclusion, swimming training effectively mitigates CS‐induced hippocampal damage and spatial memory deficits in both stressed and nonstressed rats, with the effects being more pronounced in nonstressed rats. While natural recovery allows partial restoration of hippocampal structure and function, swimming confers superior and more rapid benefits, likely through a combination of reduced neuroinflammation, enhanced neuronal survival, and possibly increased neurogenesis. Although many proposed mechanisms (e.g., BDNF, synaptic plasticity) are extrapolated from land‐based exercise, emerging evidence supports the unique role of swimming in modulating stress physiology and brain structure. Future studies should directly compare swimming with other exercise modalities and measure molecular mediators to clarify its distinct neuroprotective profile. These findings may have implications for human biology, although further studies are required.

## Disclosure

A preprint of this manuscript has been published in Research Square: doi: https://doi.org/10.21203/rs.3.rs-790123/v1 [75]. All authors have read and agreed to the published version of the manuscript.

## Conflicts of Interest

The authors declare no conflicts of interest.

## Author Contributions

Conceptualization, Mohammad Amin Safari, Maryam Koushkie Jahromi, Hadi Aligholi, Rasoul Rezaei, and Zahra Zeraatpisheh; methodology, Mohammad Amin Safari, Maryam Koushkie Jahromi, Hadi Aligholi, and Rasoul Rezaei; software, Mohammad Amin Safari, Maryam Koushkie Jahromi, and Hadi Aligholi; validation, Mohammad Amin Safari and Zahra Zeraatpisheh; formal analysis, Mohammad Amin Safari; investigation, Mohammad Amin Safari, Parisa Foroozan, and Atiyeh Separdanasab; resources, Mohammad Amin Safari and Zahra Zeraatpisheh; data curation, Mohammad Amin Safari and Maryam Koushkie Jahromi; writing, Mohammad Amin Safari and Maryam Koushkie Jahromi; writing–review and editing, Mohammad Amin Safari, Maryam Koushkie Jahromi, Hadi Aligholi, Rasoul Rezaei, and Zahra Zeraatpisheh; visualization, Mohammad Amin Safari and Maryam Koushkie Jahromi; supervision, Maryam Koushkie Jahromi; consultation, Hadi Aligholi and Rasoul Rezaei; project administration, Mohammad Amin Safari, Parisa Foroozan, and Atiyeh Separdanasab; funding acquisition, Mohammad Amin Safari.

## Funding

This research did not receive any specific grant from funding agencies in the public, commercial, or not‐for‐profit sectors.

## Data Availability

Data will be available on reasonable request from the corresponding author.
